# Children with non-central nervous system tumors treated with platinum-based chemotherapy are at risk for hearing loss and cognitive impairments

**DOI:** 10.3389/fped.2024.1341762

**Published:** 2024-03-20

**Authors:** Allison J. L’Hotta, Anne Spence, Taniya E. Varughese, Kara Felts, Susan S. Hayashi, Megan Jones-White, Emily LaFentres, Judith E. C. Lieu, Robert J. Hayashi, Allison A. King

**Affiliations:** ^1^ Brown School, Prevention Research Center, Washington University in St. Louis, St. Louis, MO, United States; ^2^ Department of Pediatrics, Division of Pediatric Hematology Oncology, Washington University in St. Louis, St. Louis, MO, United States; ^3^ Department of Otolaryngology, Division of Pediatric Otolaryngology, Washington University in St. Louis, St. Louis, MO, United States

**Keywords:** cancer survivor child, cognition, chemotherapy-related cognitive impairment, hearing loss, solid tumor

## Abstract

**Background:**

Childhood cancer survivors (CCS) with chemotherapy induced sensorineural hearing loss (SNHL) are at risk for neurocognitive impairments. The purpose of this study was to determine the relationship between SNHL and cognitive function among CCS.

**Procedure:**

Inclusion: non-CNS solid tumor diagnosis; history of platinum chemotherapy (cisplatin and/or carboplatin); 8–17 years of age; off anti-cancer treatment for ≥6 months; and English speaking. Exclusion: history of intrathecal chemotherapy, cranial radiation, or baseline neurocognitive disorder. Participants completed the NIH Toolbox Cognition Battery at enrollment. *T*-tests were used to compare participants with normal hearing to those with hearing loss and the total sample with established Toolbox normative data (mean: 50; SD: 10).

**Results:**

Fifty-seven individuals enrolled; 52 completed full cognitive testing. Participants were on average 12.2 years of age and 7.0 years since treatment completion. Twenty-one participants (40%) received cisplatin, 27 (52%) carboplatin, and 4 (8%) received both. Fifteen participants (29%) demonstrated SNHL based on the better ear. CCS, regardless of the presence or absence of SNHL, demonstrated significantly lower mean cognitive skills compared to the normative sample in attention, executive function, language- vocabulary and oral reading, processing speed, and fluid, crystallized and total composite scores (all *p *< 0.01). Participants with SNHL had significantly lower crystallized composite (vocabulary, oral reading) than those with normal hearing (41.9 vs. 47.2, *p *< 0.05, Cohen's *d* = 0.62).

**Conclusions:**

CCS at risk for platinum induced hearing loss but without cranial radiation or intrathecal chemotherapy exposure demonstrate impaired cognitive skills and those with SNHL demonstrate lower crystallized composite scores.

## Introduction

Advancements in cancer treatments over the past five decades have increased survival rates to over 85% among children diagnosed with cancer (1). As more children are surviving, there is a need to better understand the potential toxicities and side effects associated with treatment. Platinum chemotherapy agents (i.e., cisplatin, carboplatin) have contributed to increased survival rates but are also associated with the development of irreversible hearing loss (2).

Approximately 55%–60% of childhood cancer survivors (CCS) treated with platinum chemotherapy agents experience treatment-related sensorineural hearing loss (SNHL) (3, 4). SNHL is more common among children who receive cisplatin vs. carboplatin (4). Risk factors for developing SNHL in this population include younger age, higher cumulative and individual dose volume of cisplatin, and radiation to any part of the body (5).

Chemotherapy induced hearing loss is typically bilateral, although unilateral hearing loss can occur, irreversible, and affects the higher frequencies before progressing to lower frequencies. The higher frequencies include certain consonants (s, sh, f, t, z, th, h, k, p), which are frequently used in the English language, and are essential for understanding speech, especially among young children who are developing language skills (3, 6). When children are unable to perceive certain sounds due to chemotherapy-induced hearing loss, it is challenging for them to build foundational language skills (3). Hearing loss of any degree can negatively affect a variety of domains such as mental health, social-emotional development, and academic performance (2). CCS with chemotherapy-induced SNHL are at higher risk for developing neurocognitive impairments (7) and having a diagnosis of a learning disability compared to CCS without SNHL (2, 8). Significant delays in intellectual ability are common among CCS with SNHL (2, 8) and can contribute to long-term negative educational and employment outcomes including not graduating high school and experiencing unemployment (9).

Unilateral hearing loss can also be detrimental to the development of a child (10). Children with only one normal hearing ear have been shown to have lower speech-language outcomes, lower verbal intelligence, and fail grades at much higher rates compared to those with two normal hearing ears (11). In addition, cognitive fatigue, alterations in brain networks, and psychosocial issues affecting behavior and emotions can all affect the quality of life of children with unilateral hearing loss (12, 13).

Despite the link between SNHL, academic outcomes, and intellectual ability, there remains a limited understanding of the relationship between neurocognition and SNHL among a broad range of CCS. Most studies to date have attributed cognitive impairment in CCS to prior cranial radiation or intrathecal chemotherapy (14–17). The impact of SNHL influencing neurocognitive performance may be obscured by these well-known risk factors for cognitive impairment (2). We specifically strived to eliminate known risk factors for cognitive impairment to better assess the impact of hearing loss on cognition. This pilot study seeks to address the gap in our understanding of how chemotherapy-induced hearing loss impacts cognition among children with non-CNS tumors and no head or neck radiation exposure.

The primary objective of the current pilot study was to examine the relationship between SNHL and cognitive function among CCS with non-CNS tumors. We hypothesized that SNHL would negatively affect cognitive function in CCS with non-CNS tumors. Secondary study objectives included comparing cognitive function of CCS to established normative sample data and to explore differences in cognition based on the degree of hearing impairment (e.g., no hearing loss vs. mild vs. severe hearing loss). We expected that children with a greater degree of hearing impairment (i.e., higher SIOP score) would demonstrate lower cognitive scores, demonstrating a greater degree of cognitive impairment.

## Methods

This was a cross-sectional exploratory pilot study of CCS followed in the Division of Hematology and Oncology at St. Louis Children's Hospital and St. Louis Children's Specialty Care Center. Due to the exploratory pilot nature of the study, and the absence of published correlations on study outcomes, an *a priori* power analysis was not conducted. The institutional review board and protocol review and monitoring committee at Washington University School of Medicine in St. Louis approved study procedures (IRB # 201807077, initial approval date: September 5, 2018).

### Participants

CCS with non-CNS malignancies were identified by the managing care team through medical chart review and provider knowledge of the individual's prior treatment. A member of the pediatric hematology/oncology program or an established study team delegate approached potential participants and their parent/guardian about the study during a routine outpatient visit. Our institution has a long-standing audiology ototoxicity monitoring program which obtains baseline hearing assessments and serial monitoring in CCS exposed to platinum chemotherapy to assist in the early identification of individuals with hearing loss. The type of hearing test completed was based on a child's age, overall health and functional status. Patients who were at risk for hearing loss continued to be monitored following completion of therapy. Consequently, the audiograms obtained were part of their routine standard-of-care follow-up and management with the Audiology Department at St. Louis Children's Hospital to assess for development and progression of SNHL. The existence of this critical care pathway supported participant enrollment.

Eligible individuals were diagnosed with a non-CNS pediatric solid tumor with history of ototoxic chemotherapy (cisplatin and/or carboplatin), 8–17 years old at the time of study participation, off all anti-cancer treatment for a minimum of 6 months, had an audiogram obtained with good-to-fair reliability within 1 year of enrollment, and English speaking. Individuals were excluded from participation if they had a history of a CNS tumor, intrathecal chemotherapy, cranial radiation, baseline neurocognitive or psychological disorder, or if the parent and/or patient were unable to read English. All participants ages 8–17 years provided assent; a parent/guardian also provided informed consent. Participants received a $50 retail gift card following the completion of all study procedures.

### Data collection/procedures

Following informed consent, participants completed a one-time testing session in a private clinic room on the day of enrollment. Participants completed the National Institutes of Health (NIH) Toolbox Cognition Battery in-person on an iPad with a trained research team member who was not directly involved in the child's clinical care. Expected time to complete the measure is approximately 30 min (18).

The *NIH Toolbox Cognition Battery* is a rigorously developed standardized measure of cognition for individuals 7 years of age and older. Cognitive domains assessed include: attention, executive function, episodic memory, working memory, language (vocabulary and oral reading), and processing speed (19). Three composite scores are calculated: total composite includes all test subdomains, crystallized composite includes the two language subdomains, and fluid composite combines attention, executive function, memory, and processing speed. Scores are reported as fully-corrected *T*-scores, corrected for a child's age, gender, education, and race/ethnicity, factors that can lead to meaningful differences in scores (20). Normative sample scores for the Cognition Battery have a mean *T*-score of 50 and standard deviation of 10 (20). Data were automatically scored through the NIH Toolbox application and were transferred to REDCap electronic data capture tools (21, 22) hosted at Washington University in St. Louis for storage.

#### Audiogram

All participants had a routine audiogram as part of standard care. Normal hearing was defined as thresholds ≤20 dB HL across frequencies. SNHL was defined as air conduction pure tone thresholds at any frequency ≥25 dB HL accompanied by bone conduction thresholds within 10 dB of the air conduction threshold or a hearing loss greater than a SIOP 0. Asymmetric hearing loss was defined as a difference of ≥20 dB at one frequency, or a difference ≥15 dB difference at two or more frequencies between ears. Unilateral hearing loss indicates SNHL in one ear and normal hearing in the other ear (23).

Audiogram results were categorized according to the International Society of Pediatric Oncology (SIOP) ototoxicity grading scale (6). The SIOP scale is the recommended standard for monitoring hearing during cancer treatment and is an easy-to-use, clinically applicable scale for classifying ototoxic hearing loss (24). Use of the SIOP grading scale supports comparison of findings across institutions. SIOP grades range from zero to four where zero indicates normal hearing sensitivity and higher grades indicates a greater level of SNHL. For the purposes of this study, a SIOP grade 1 or 2 indicated a mild hearing impairment and a SIOP grade 3 or 4 indicated a severe hearing impairment. See Supplementary Figure S1 for detailed SIOP grading criteria.

#### Medical record extraction

Data extracted from the electronic medical record included demographic characteristics (current age and age at diagnosis, gender, race), cancer diagnosis and treatment (ototoxic chemotherapy received, cumulative dose) and amplification use (date fitted for hearing aid, other hearing assistive technologies).

#### Statistical analysis

Descriptive statistics were compiled for demographic characteristics and to summarize outcome measures. Based on methods used in prior research (4), SIOP grade of the better hearing ear was used to determine the severity of the participant's SNHL for the primary analyses. A secondary analysis was conducted in which SNHL was classified based on the participant's worse hearing ear due to the known detrimental impact of unilateral hearing loss including speech and language delays, difficulty localizing sound, and difficulty understanding speech in noise (25). To evaluate differences in cognition between participants with normal hearing (SIOP grade 0) in the better hearing ear to those with SNHL (SIOP grades 1–4), independent samples *t*-tests were used with a significance level of 0.05. One-sample *t*-test compared average NIH toolbox cognition scores for the total sample with the established normative sample mean of 50; Cohen's d effect sizes are reported. Odds ratios were calculated to measure the association between SNHL and cognitive impairment.

Pearson correlations I were run to evaluate the relationships between cognitive outcomes and demographic and treatment characteristics (age at diagnosis and testing, time since diagnosis, time since treatment completion, cumulative cisplatin [mg/m^2^], carboplatin [mg/m^2^], and radiation dose [cGy]). To investigate differences in cognition based on cancer type, one-way ANOVA was used. An exploratory analysis was conducted via one-way ANOVA to assess differences in cognition between participants with no hearing loss vs. mild hearing loss vs. severe hearing loss in their better hearing ear. These methods were selected because the cognitive outcome data were normally distributed for the sample. Missing data were assumed to be missing not at random (i.e., child was fatigued and did not complete testing) and/or were due to one participant having been previously treated at an outside institution, and therefore these data were left as missing.

## Results

**Figure 1:**
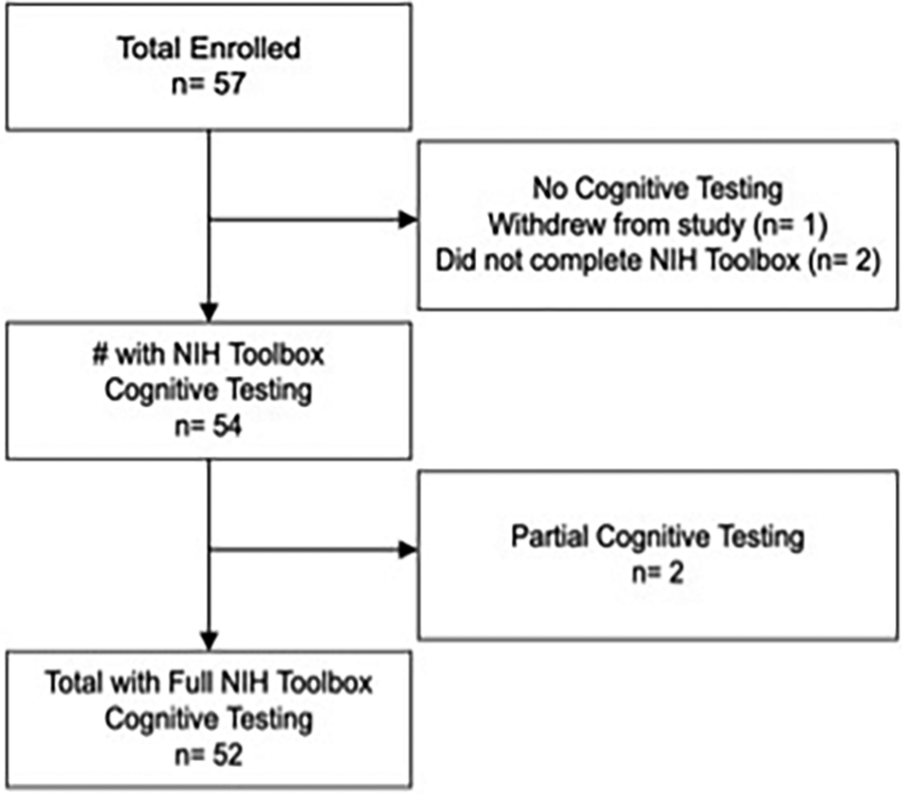
Participant flow.

Fifty-seven individuals enrolled in the study. Full cognitive testing was available for 52 participants (Figure 1). Average age at study entry was 12.2 years (range: 8–17). Participants were on average 7.0 years (range: 8 months to 16 years) post completion of anti-cancer therapies. Neuroblastoma (31%), germ cell tumor (25%), and retinoblastoma (20%) were the most common diagnoses. Twenty-one participants (40%) received cisplatin, 27 (52%) received carboplatin and 4 (8%) received both; the average cumulative dose for each agent is reported in Table 1. Based on audiogram assessment, 15 (29%) participants experienced bilateral treatment-related hearing loss, while an additional 2 (4%) participants experienced unilateral treatment-related hearing loss, with normal hearing in their better ear (SIOP = 0). Therefore, when evaluating SNHL based on the better hearing ear, 15 (29%) demonstrated hearing loss, and when evaluating SNHL based on the worse hearing ear, 17 (33%) demonstrated hearing loss. Two individuals who completed only partial cognitive testing also had SNHL; these participants were excluded from analyses due to incomplete data. Seven participants (13%) had been previously fit with bilateral hearing aids and two (4%) with a unilateral hearing aid. All participants who were previously fit with hearing aids were required to wear them during study measures. Data on SIOP grade are presented in Table 1.

**Table 1:** Participant demographic, treatment, and hearing characteristics (*n* = 52).

Characteristic	*n* (%) or mean (range; SD)				
Gender					
Male	27 (51.9)				
Female	25 (48.1)				
Age in years (mean)	12.19 (8–17; SD 2.90)				
Age in years at diagnosis	3.77 (0–15; SD 4.29)				
Time in years since diagnosis	7.92 (1–16; SD 4.52)				
Time in years since treatment completion	7.08 (0–16; SD 4.66)				
Race					
Caucasian	48 (92.3)				
African American	2 (3.8)				
Asian	1 (1.9)				
Other	1 (1.9)				
Diagnosis					
Neuroblastoma	16 (31.5)				
Germ cell tumor	13 (24.1)				
Retinoblastoma	10 (18.5)				
Osteosarcoma	4 (7.4)				
Wilms tumor	4 (7.4)				
Hepatoblastoma	4 (7.4)				
Clear cell carcinoma of kidney	1 (3.7)				
Treatment					
Mean cumulative cisplatin dose (mg/m^2^) (*n* = 24^a^, dose unavailable for one participant)	468.90 (134.15–800; SD 170.42)				
Mean cumulative carboplatin dose (mg/m^2^) (*n* = 31)	2,620.82 (560–9,350; SD 1,670.01)				
Received radiation (*n* = 51^a^, radiation data unavailable for one participant)	11 (21.2)				
Location of radiation (multiple sites allowed)					
Abdomen	10 (19.2)				
Chest	3 (5.8)				
Groin	1 (1.9)				
Pelvis	2 (3.8)				
Not applicable	41 (76.9)				
Mean total dosage of radiation (*n* = 11)	2,607.27 (1,080–4,200; SD 1,038.32)				
Hearing					
SIOP grade	Grade 0	Grade 1	Grade 2	Grade 3	Grade 4
Better hearing ear	37 (71.2)	4 (7.7)	5 (9.6)	5 (9.6)	1 (1.9)
Worse hearing ear	35 (67.3)	2 (3.9)	5 (9.6)	9 (17.3)	1 (1.9)
Asymmetric hearing	8 (15.4)				
Symmetric hearing	44 (84.6)				
Bilateral hearing loss	15 (28.8)				
Unilateral hearing loss	2 (3.8)				
Hearing aid use (*n* = 9)					
Bilateral	7 (77.8)				
Unilateral	2 (22.2)				

NH, normal hearing; SNHL, sensorineural hearing loss; SIOP, International Society of Pediatric Oncology.

^a^ Missing data for one participant treated at an outside institution; detailed treatment records were unavailable.

### Differences in cognition between total sample and normative data

The total sample, including CCS with and without SNHL in their better hearing ear, had significantly lower mean cognitive skills in the domains of attention (*p* < 0.001), executive function (*p* = 0.005), vocabulary (*p* = 0.008), oral reading (*p* = 0.001), processing speed (*p* = 0.007), fluid composite (*p* < 0.001), crystallized composite (*p* = 0.001), and total composite (*p* < 0.001) compared to normative data (mean: 50, SD: 10; Table 2). Medium effect sizes were noted in multiple domains including language-oral reading and all composite scores (fluid, crystallized and total). A large effect size was observed for attention (Cohen's *d* = −1.21). The total study sample did not differ from normative data on the subdomains of episodic (*p* = 0.198) or working memory (*p* = 0.852).

**Table 2:** Differences in cognition between CCS and population normative means.

Cognitive domain	Total sample mean (SD) *n* = 52	Effect size (Cohen's *d*)	*p*-value
Attention	40.7 (7.7)	−1.21	<0.001*
Executive function	45.9 (10.1)	−0.41	0.005*
Episodic memory	52.2 (12.3)	0.18	0.198
Language- vocabulary	46.6 (9.0)	−0.38	0.008*
Working memory	49.8 (8.9)	−0.03	0.852
Language-oral reading	46.3 (7.7)	−0.49	0.001*
Processing speed	44.4 (14.2)	−0.39	0.007*
Fluid composite	44.1 (11.4)	−0.52	<0.001*
Crystallized composite	45.7 (8.9)	−0.49	0.001*
Total composite	43.7 (10.3)	−0.62	<0.001*

* Indicates *p* ≤ 0.01.

### Differences in cognition between CCS with SNHL vs. normal hearing

Participants with SNHL in their better ear scored significantly lower than those with normal hearing in at least one ear on the crystallized composite score, which includes vocabulary and oral reading skills (SNHL mean of 41.9, SD 10.7 vs. 47.2, SD 7.7 for those with normal hearing; Table 3). A moderate effect size was found for crystallized composite scores (Cohen's *d* = 0.62). No other significant differences were observed between CCS with SNHL compared to those with normal hearing in at least one ear. Among participants with SNHL based on their worse hearing ear, including those who have symmetric SIOP grading in both ears and those whose SIOP grades between ears may be different, those with SNHL scored significantly lower on episodic memory (mean: 47.9, SD: 10.7) compared to those with normal hearing (mean: 54.8, SD: 12.4), *p* = 0.03 (Supplementary Table S1).

**Table 3:** Differences in cognition between CCS with sensorineural hearing loss vs. normal hearing in better ear.

Cognitive domain	Sensorineural hearing loss mean (SD) *n* = 15	Normal hearing mean (SD) *n* = 37	Effect size (Cohen's *d*)	*p*-value
Attention	41.1 (9.3)	40.5 (7.1)	−0.08	0.787
Executive function	46.5 (13.1)	45.6 (8.9)	−0.09	0.773
Episodic memory	48.1 (11.0)	53.9 (12.6)	0.48	0.122
Language- vocabulary	43.5 (9.9)	47.8 (8.4)	0.49	0.113
Working memory	46.8 (8.1)	51.0 (9.0)	0.48	0.127
Language-oral reading	43.3 (9.4)	47.5 (6.6)	0.56	0.720
Processing speed	45.3 (15.3)	44.1 (13.9)	−0.09	0.776
Fluid composite	42.3 (12.8)	44.8 (10.9)	0.22	0.475
Crystallized composite	41.9 (10.7)	47.2 (7.7)	0.62	0.049*
Total composite	40.0 (10.7)	45.1 (9.9)	0.51	0.104

* Indicates *p* ≤ 0.05.

### Differences in cognition based on level of hearing impairment

Exploratory analyses did not reveal any significant differences in cognitive skills between individuals with normal hearing in at least one ear (SIOP 0) and those with mild (SIOP 1–2) or more severe (SIOP 3–4) SNHL (Supplementary Table S2).

### Relationship between cognition, disease, and treatment characteristics

There were no statistically significant correlations between cognition and age at diagnosis or cumulative carboplatin dose (mg/m^2^). There were weak relationships between time since diagnosis and time since treatment completion with total composite scores (*r* = 0.301, *p* = 0.030; *r* = 0.297, *p* = 0.038, respectively). Age at time of testing and language-oral reading (*r* = 0.325, *p* = 0.019), processing speed (*r* = 0.318, *p* = 0.021), fluid composite (*r *= 0.347, *p* = 0.012), total composite (*r* = 0.298, *p* = 0.032) scores were all significantly and positively correlated. Cumulative cisplatin dose (mg/m^2^) was significantly negatively associated with language-oral reading (*r* = −0.482, *p* = 0.017) and crystallized composite scores (*r* = −0.437, *p* = 0.033). Radiation dose (cGy) was significantly negatively associated with language-oral reading (*r* = −0.742, *p* = 0.009). There were no statistically significant differences in cognitive outcome based on cancer type nor SIOP grade based on treatment exposure.

### Proportion with impaired cognition

The number of participants with impaired cognitive composite scores, as measured by the NIH Toolbox Cognition Battery, is available in Supplementary Table S3. Notably, a larger proportion of participants with SNHL in both ears demonstrated a higher degree of cognitive impairment compared to those patients with at least one normal hearing ear. For example, 24% with SNHL scored 2 standard deviations or more below the mean on fluid composite scores compared to 11% with normal hearing in at least one ear. Similarly, on the total composite, 24% with SNHL compared to 0% with normal hearing in at least one ear scored 2 standard deviations or more below the mean. Differences are also noted in the proportion with crystallized and total composite scores more than 1 standard deviation below the mean (Supplementary Table S3). Exploratory analyses of odds ratios revealed the only significant association between hearing loss and cognitive deficits was in the crystallized cognition composite (OR: 9.43, 95% CI: 2.2–40.2, *p *= 0.002).

## Discussion

In this study, CCS not traditionally considered at risk for cognitive impairments based on their disease and treatment history (i.e., non-CNS tumors, no cranial radiation or intrathecal chemotherapy) demonstrated significantly impaired cognitive skills compared to population normative scores. Nearly 2 of 5 participants (39%) demonstrated impaired cognition based on the NIH Toolbox total composite score. This aligns with findings from a large database study by Phillips et al. in which 35% of CCS demonstrated neurocognitive dysfunction (26). However, in contrast to that report, our study excluded CCS with CNS tumors.

The entire study cohort demonstrated cognitive challenges regardless of hearing status, suggesting that platinum-based chemotherapy may lead to cognitive impairment. Our study also confirms prior reports that language skills (vocabulary, oral reading) are significantly more impaired in CCS with SNHL compared to those with normal hearing (4). Given these increased risks, there is a need to reevaluate guideline recommendations for longitudinal neurocognitive testing CCS to provide testing to a broader population.

Children's Oncology Group (COG) and National Comprehensive Cancer Network (NCCN) guidelines include recommendations for neurocognitive testing among CCS. However, these guidelines recommend neurocognitive testing almost exclusively among children with treatment exposures that put them at the highest risk for neurocognitive impairments (27, 28). For populations considered at lower risk for impairment, recommendations are vague, such as evaluate with neuropsychological assessment “as indicated” or if a problem is suspected (27, 29, 30). Such recommendations often rely on patients and families raising concerns, which is worrisome due to the subtle nature of many cancer-related cognitive impairments. Additionally, relying on family report may favor more highly educated families and widen disparities.

Management of cognitive and hearing function following treatment for cancer necessitates an interdisciplinary approach with collaboration between oncology, audiology, and cognitive rehabilitation providers (e.g., neuropsychology, occupational therapy, speech language pathology). To support screening in a busy clinical oncology setting, workflows that include efficient screenings for hearing loss and cognition (e.g., NIH Toolbox) should be identified. The goal in prospectively evaluating hearing and cognition is to facilitate early referral and intervention. CCS must be connected with evidence-based rehabilitation interventions and other academic or behavioral supports based on testing recommendations (27, 31). Developing, testing, and implementing cognitive rehabilitation interventions in clinical practice must be prioritized given the paucity of effective cognitive interventions for CCS (32, 33). While the current body of evidence for cognitive interventions among CCS is small, clinicians and researchers can consider the use of physical activity and computerized cognitive training programs for CCS (34, 35).

Larger longitudinal studies are needed to further clarify the scope and nature of cognitive impairments in CCS with hearing loss from platinum-based chemotherapy regimens. The lack of a baseline cognitive assessment limited our ability to understand changes that occurred within individuals following treatment initiation. Prospective longitudinal studies would allow us to examine how early intervention and consistent amplification use further impacts cognitive outcomes in CCS with hearing loss. The small sample size in this pilot study may explain why we did not identify greater differences in cognitive function between those with SNHL vs. normal hearing. While we did identify a significantly higher risk for impaired crystalized cognition among those with SNHL, the confidence interval was wide. An *a priori* power analysis was not completed due to the pilot nature of the study and the absence of published correlations between study outcomes at study initiation. These data can serve as preliminary data for future, larger scale longitudinal studies, which should consider inclusion of a control group comparator. Larger studies would support more detailed analyses of the influence of socioeconomic and treatment-related factors on hearing and cognitive outcomes. Future studies should also include collection of qualitative data to develop a more in-depth understanding of this complex problem.

Multiple opportunities exist to explore the mechanisms underlying the cognitive deficits experienced by children who were treated with chemotherapy and have hearing loss. Future studies are needed to explore the mechanisms of these deficits. Prior animal studies have demonstrated that cisplatin is associated with mitochondrial damage, cognitive changes, and markers of inflammation (36, 37). We are unaware of studies that are specific to cortical pathway disruption among children with cancer. However, Lieu and colleagues (2017) have demonstrated children with unilateral hearing loss demonstrate different functional brain network connections compared to normal hearing siblings (13).

This study highlights the need for further research on the impact of SNHL on cognition in CCS. Additional efforts examining a larger sample size of CCS treated with platinum chemotherapy are needed. Furthermore, study findings raise concerns that exposure to platinum chemotherapy may be a risk factor for cognitive impairment.

## Data Availability

The raw data supporting the conclusions of this article will be made available by the authors, without undue reservation.
