# Supplementary material for: Children with non-central nervous system tumors treated with platinum-based chemotherapy are at risk for hearing loss and cognitive impairments
Source: Front Pediatr. 2024 Mar 20;12:1341762. doi: 10.3389/fped.2024.1341762 (PMC10987805; doi:10.3389/fped.2024.1341762)
Supplement: Supplementary file 1 [file Table1.docx]

Supplemental Table 1. Differences in cognition between CCS with sensorineural hearing loss in worse hearing ear versus normal hearing in worse hearing ear

| Cognitive Domain | Sensorineural Hearing Loss in Worse Ear  Mean (SD)  n= 17 | Normal Hearing in Worse Ear  Mean (SD)  n= 35 | Effect Size (Cohen’s d) | p-value |
| --- | --- | --- | --- | --- |
| Attention | 41.9 (10.3) | 40.1 (6.2) | -0.23 | 0.515 |
| Executive Function | 47.7 (13.8) | 45.0 (7.9) | -0.26 | 0.476 |
| Episodic Memory | 47.9 (10.7) | 54.8 (12.4) | 0.66 | 0.030* |
| Language- Vocabulary | 44.9 (11.8) | 47.4 (7.3) | 0.28 | 0.349 |
| Working Memory | 48.5 (9.2) | 50.4 (8.8) | 0.22 | 0.469 |
| Language-Oral Reading | 44.1 (10.1) | 47.3 (6.1) | 0.42 | 0.241 |
| Processing Speed | 46.6 (15.8) | 43.4 (13.4) | -0.22 | 0.452 |
| Fluid Composite | 43.5 (13.7) | 44.3 (10.3) | 0.08 | 0.798 |
| Crystallized Composite | 43.2 (12.4) | 46.9 (6.5) | 0.41 | 0.272 |
| Total Composite | 41.6 (13.3) | 44.7 (8.5) | 0.30 | 0.395 |
