# Supplementary material for: Children with non-central nervous system tumors treated with platinum-based chemotherapy are at risk for hearing loss and cognitive impairments
Source: Front Pediatr. 2024 Mar 20;12:1341762. doi: 10.3389/fped.2024.1341762 (PMC10987805; doi:10.3389/fped.2024.1341762)
Supplement: Supplementary file 2 [file Table2.docx]

Supplemental Table 2. Differences in cognition based on level of hearing impairment

| Cognitive Domain | Normal Hearing Mean (SD)  n= 37 | Mild Hearing Impairment Mean (SD) n=9 | More Severe Hearing Impairment Mean (SD) n=6 | F statistic | p-value |
| --- | --- | --- | --- | --- | --- |
| Attention | 40.5 (7.1) | 37.4 (5.2) | 46.7 (11.8) | 2.79 | 0.071 |
| Executive Function | 45.6 (8.9) | 45.4 (10.4) | 48.2 (17.3) | 0.17 | 0.847 |
| Episodic Memory | 53.9 (12.6) | 44.9 (5.7) | 52.8 (15.5) | 2.03 | 0.143 |
| Language- Vocabulary | 47.8 (8.4) | 45.2 (11.9) | 40.8 (5.8) | 1.74 | 0.186 |
| Working Memory | 51.0 (9.0) | 46.3 (10.0) | 47.5 (4.6) | 1.21 | 0.306 |
| Language-Oral Reading | 47.5 (6.6) | 44.1 (11.0) | 42.0 (7.0) | 1.81 | 0.175 |
| Processing Speed | 44.1 (13.9) | 40.4 (12.4) | 52.7 (17.4) | 1.41 | 0.255 |
| Fluid Composite | 44.8 (10.9) | 37.9 (10.9) | 48.8 (13.3) | 2.00 | 0.146 |
| Crystallized Composite | 47.2 (7.7) | 43.2 (13.1) | 39.8 (6.2) | 2.29 | 0.112 |
| Total Composite | 45.1 (9.9) | 38.3 (12.0) | 42.5 (8.6) | 1.66 | 0.200 |

 Mild hearing impairment group includes SIOP Grades 1 or 2 in the participant’s better hearing ear. More severe hearing impairment group includes SIOP Grades 3 or 4 in the participant’s better hearing ear.
