# Supplementary material for: Children with non-central nervous system tumors treated with platinum-based chemotherapy are at risk for hearing loss and cognitive impairments
Source: Front Pediatr. 2024 Mar 20;12:1341762. doi: 10.3389/fped.2024.1341762 (PMC10987805; doi:10.3389/fped.2024.1341762)
Supplement: Supplementary file 3 [file Table3.docx]

Supplemental Table 3. Proportion with impaired NIH Toolbox Cognition Battery composite scores

|  | Sensorineural hearing loss (n= 15) | Normal Hearing (n=37) | Total Sample  (n= 52) |
| --- | --- | --- | --- |
|  | n (%) | | |
| Fluid composite, ≤ 40 T > 30 | 3 (20) | 11 (30) | 14 (27) |
| Fluid composite, T ≤ 30 | 4 (27) | 4 (11) | 8 (15) |
| Crystallized composite, ≤ 40 T > 30 | 6 (40) | 4 (11) | 10 (19) |
| Crystallized composite, T ≤ 30 | 2 (13) | 0 (0) | 2 (4) |
| Total composite, ≤ 40 T > 30 | 2 (13) | 14 (38) | 16 (31) |
| Total composite, T ≤ 30 | 4 (27) | 0 (0) | 4 (8) |

Note: ≤ 40 T > 30 indicates scores more than 1, but less than 2, standard deviations below the mean and T ≤ 30 indicates scores 2 standard deviations or more below the mean.
