# Supplementary material for: Children with non-central nervous system tumors treated with platinum-based chemotherapy are at risk for hearing loss and cognitive impairments
Source: Front Pediatr. 2024 Mar 20;12:1341762. doi: 10.3389/fped.2024.1341762 (PMC10987805; doi:10.3389/fped.2024.1341762)
Supplement: Supplementary file 4 [file Image1.pdf]

Supplemental Figure 1. SIOP Boston Ototoxicity Scale

Grade	Parameters
0	Less than or equal to 20 dB HL at all frequencies
1	> 20 dB HL (i.e., 25 dB HL or greater) SNHL above 4,000 Hz (i.e., 6 or 8 kHz)
2	> 20 dB HL SNHL at 4,000 Hz and above
3	> 20 dB HL SNHL at 2,000 Hz or 3,000 Hz and above
4	> 40 dB HL (i.e., 45 dB HL or more) SNHL at 2,000 Hz and above
